# Neural correlates of maintaining one’s political beliefs in the face of counterevidence

**DOI:** 10.1038/srep39589

**Published:** 2016-12-23

**Authors:** Jonas T. Kaplan, Sarah I. Gimbel, Sam Harris

**Affiliations:** 1 Brain and Creativity Institute and Department of Psychology, University of Southern California Los Angeles, CA, 90089, USA; 2 Project Reason Los Angeles, CA, USA

## Abstract

People often discount evidence that contradicts their firmly held beliefs. However, little is known about the neural mechanisms that govern this behavior. We used neuroimaging to investigate the neural systems involved in maintaining belief in the face of counterevidence, presenting 40 liberals with arguments that contradicted their strongly held political and non-political views. Challenges to political beliefs produced increased activity in the default mode network—a set of interconnected structures associated with self-representation and disengagement from the external world. Trials with greater belief resistance showed increased response in the dorsomedial prefrontal cortex and decreased activity in the orbitofrontal cortex. We also found that participants who changed their minds more showed less BOLD signal in the insula and the amygdala when evaluating counterevidence. These results highlight the role of emotion in belief-change resistance and offer insight into the neural systems involved in belief maintenance, motivated reasoning, and related phenomena.

Few things are as fundamental to human progress as our ability to arrive at a shared understanding of the world. The advancement of science depends on this, as does the accumulation of cultural knowledge in general. Every collaboration, whether in the solitude of a marriage or in a formal alliance between nations, requires that the beliefs of those involved remain open to mutual influence through conversation. Data on any topic—from climate science to epidemiology—must first be successfully communicated and *believed* before it can inform personal behavior or public policy. Viewed in this light, the inability to change another person’s mind through evidence and argument, or to have one’s own mind changed in turn, stands out as a problem of great societal importance. Both human knowledge and human cooperation depend upon such feats of cognitive and emotional flexibility.

It is well known that people often resist changing their beliefs when directly challenged, especially when these beliefs are central to their identity^1^,^2^,^3^,^4^,^5^,^6^. In some cases, exposure to counterevidence may even increase a person’s confidence that his or her cherished beliefs are true^7^,^8^. Although neuroscientists have begun to study some of the social aspects of persuasion^9^ and motivated reasoning^10^, little research is aimed directly at understanding the neural systems involved in protecting our most strongly held beliefs against counterevidence.

One model of belief maintenance holds that when confronted with counterevidence, people experience negative emotions borne of conflict between the perceived importance of their existing beliefs and the uncertainty created by the new information^11^,^12^,^13^,^14^. In an effort to reduce these negative emotions, people may begin to think in ways that minimize the impact of the challenging evidence: discounting its source, forming counterarguments, socially validating their original attitude, or selectively avoiding the new information^15^. The degree to which such rationalization occurs depends upon several factors, but the personal significance of the challenged belief appears to be crucial. Specifically, beliefs that relate to one’s social identity are likely to be more difficult to change^16^,^17^,^18^,^19^.

Based on this model, predictions can be made about the neural systems that govern resistance to belief change. First, resistance to evidence may entail disengagement from external reality and increased inward focus. The brain’s default mode network (DMN), including posterior and anterior midline structures and the lateral inferior parietal lobes, appears to support these psychological processes^20^,^21^. Identity-related beliefs might also invoke internal models of the self, a form of cognition that is associated with increased activity within the DMN^22^,^23^. Second, if resistance to belief change is partly motivated by negative emotion, having one’s beliefs contradicted may produce activity in associated regions of the brain, such as the amygdala, the insular cortex, and other structures involved in emotion regulation.

In this study, we performed functional MRI to measure the brain activity of 40 individuals with strong political views as they encountered arguments against their beliefs. All the subjects were self-identified as political liberals of deep conviction. Inside the fMRI scanner, participants saw a series of statements they previously indicated strongly believing, followed by several challenging counterarguments. After participants read all five counterarguments, the original statement was shown again and they reported their post-challenge belief strength. The difference between pre-scan and post-challenge ratings was used as a measure of belief change. In order to compare high belief persistence to low belief persistence, in one condition we challenged strongly held political beliefs, and in another condition we challenged strongly-held non-political beliefs. While the non-political beliefs were just as strongly held according to the participants who held them, we did not expect these beliefs to be defended with the same vigor.

We predicted that the political condition would result in less belief change than the non-political condition, and that resisting challenges to political beliefs would be associated with increased activity in brain systems involved in contemplating identity and internally-focused cognition. Furthermore, we predicted that there would be a relationship between activity in emotion-related brain structures and individual differences in persuadability. We also sought to identify brain activity that correlated with the strength with which specific beliefs were maintained in our sample.

## Materials and Methods

### Participants

Forty healthy participants with no history of psychological or neurological disorders were recruited from the University of Southern California community and the surrounding Los Angeles Area (mean age: 24.30 ± 0.92 years, range: 18–39 years, 20 male). All participants were right-handed according to their own report. Subjects were paid $20 per hour for their participation and gave informed consent. All experimental protocols were approved by the Institutional Review Board of the University of Southern California and procedures were carried out in accordance with the approved guidelines. All participants had spent the majority of their life living in the United States and spoke fluent English, identified themselves as politically liberal, and had strongly held political and non-political beliefs. Specifically, participants answered a screening questionnaire in which they were asked about their political identification. On the question “Do you consider yourself a political person?” answers ranged on a scale from 1 (not at all) to 5 (very much). Participants were only included if they answered at least a 4 on this question. For the question “Which of the following describes your political self-identification?” answers ranged from 1 (strongly liberal) to 7 (strongly conservative) and participants were only included if they answered 1 or 2. Additionally, participants rated their agreement with several political and non-political statements and were only included in the experiment if they strongly agreed with at least 8 political and 8 non-political statements. Of 116 people who responded to our advertisements, 98 met the requirements for age, handedness, and political orientation. From those 98 people, 40 subjects met the requirements for strongly agreeing to at least 8 statements in each category.

### Stimuli

In this experiment, each participant read 8 political statements and 8 non-political statements with which they had previously indicated strong agreement. Each statement was followed by 5 challenges. Each challenge was a sentence or two that provided a counter-argument or evidence against the original statement.

The 8 political statements for each participant were drawn from a pool of 9 political statements. These statements concerned policy issues on which we expected predictable, identity-consistent positions from our subjects, such as “Abortion should be legal” and “Taxes on the wealthy should generally be increased”. The statements can be found in full in Table S3. The 8 non-political statements were drawn from a pool of 14 non-political statements. The pool of non-political statements was larger because while the inclusion criteria guaranteed the participants would hold certain political beliefs, they did not guarantee belief in any specific non-political statement. The non-political statements covered a wide range of topics including health (e.g. “Taking a daily multivitamin improves ones health”), education (e.g. “A college education generally improves a person’s economic prospects”), and history (e.g. “Thomas Edison invented the light bulb”).

Each political and non-political statement was associated with 5 challenges. In order to be as compelling as possible, the challenges often contained exaggerations or distortions of the truth. For instance, one challenge to the statement “The US should reduce its military budget” was “Russia has nearly twice as many active nuclear weapons as the United States”. In truth, according to statistics published by the Federation of American Scientists: Status of World Nuclear Forces (www.fas.org) in 2013, Russia has approximately 1,740 active nuclear warheads, while the United States has approximately 2,150. Examples of the challenges are provided in Table S4.

The political and non-political statements did not differ in number of words (political: 11.22 ± 1.51, non-political: 11.14 ± 1.33, *p* = 0.97), letters (political: 59.33 ± 7.71, non-political: 58.64 ± 6.04, *p* = 0.94), or Flesch reading ease (political: 60.7 ± 17.89, non-political: 48.3 ± 28.64, *p* = 0.26)^24^. The political and non-political challenges also did not differ in number of words (political: 20.44 ± 2.83, non-political: 18.92 ± 1.13, *p* = 0.15), letters (political: 104.18 ± 15.50, non-political: 96.24 ± 6.08, *p* = 0.15), or Flesch reading ease (political: 53.9 ± 21.02, non-political: 55.74 ± 19.11, *p* = 0.65).

Because we were interested in brain structures that are known to respond to social and mental stimuli, we used a word counting method to count the frequency of social and cognitive words within the stimuli. This technique is similar to linguistic inquiry word counting (LIWC^25^), but used the open-source software tool TACIT^26^ version 1.0.0 in combination with the LIWC 2007 dictionary to count words in the social and cognitive process categories. We found that such words were infrequent in our stimuli, and similar across the two categories (social words occurred with a frequency of 5.07% in the political challenges and 5.35% in the non-political challenges; cognitive words occurred with a frequency of 10.14% in the political challenges and 11.9% in the non-political challenges).

### Experimental Procedure

In preparation for the study, participants filled out a survey of demographic information, answered questions about their political and religious affiliations, and indicated the degree to which they agreed with political and non-political statements. Only statements for which participants chose 6 or 7 (where 1 was strongly disagree and 7 was strongly agree) were used during their scan. If a given subject strongly believed more than 8 statements in a category, the statements were chosen for that subject as follows: first, preference was given to more strongly held beliefs (7 vs. 6). Second, all else being equal, preference was given for statements that were not as commonly believed, in order to balance the frequency of statements in the experiment.

When participants arrived for their fMRI scan, they were given instructions and were given the opportunity to ask questions of the experimenter. After the instructions, they performed a practice task, which consisted of a shortened version of one trial of the experiment using the statement “Cats make better pets than dogs”. followed by three challenges to that statement. Following the practice task, participants underwent BOLD fMRI. For each participant there were 4 belief-challenging scans (420 seconds each). During the belief-challenging scans, each statement was presented for 10 seconds, followed by a variable delay of 4–6 seconds. Participants were instructed to press a response button when they had read and understood the statement. Five challenges to the original statement were then presented, each for 10 seconds. Again, participants pressed a response button when they had read and understood the challenge. After all five challenges had been presented, the original statement was presented again and participants had 12 seconds to rate their strength of belief in the statement. The participant indicated his or her response via a button press on an MRI-compatible button box held in the right hand. They pressed buttons to move a cursor left and right along a Likert scale to indicate the strength of their belief on a scale from 1 (strongly disbelieve) to 7 (strongly believe). The cursor started in the middle position of the scale. Two political and two non-political statements were presented in each of the four fMRI scans. The order of these conditions was randomized within each scan, and the statements within each condition were assigned random positions within the experiment for each subject. The temporal structure of the trials and runs is depicted in Fig. S1.

Following the fMRI session, participants filled out a short questionnaire. They were asked to rate how credible they found the challenges in general, and how challenging they were to their beliefs. Participants did not make separate ratings per item or per category, but rather answered these questions about their reaction to the stimulus set in general. During the debriefing, subjects were given a packet of sourced information which detailed the truth of each challenge they read inside the scanner and provided resources on where to find further information.

### MRI Scanning

Imaging was performed using a 3T Siemens MAGNETON Trio System with a 12-channel matrix head coil at the Dana and David Dornsife Neuroscience Institute at the University of Southern California. Functional images were acquired using a gradient-echo, echo-planar, T2*-weighted pulse sequence (TR = 2000 msec, one shot per repetition, TE = 25 msec, flip angle = 90°, 64 × 64 matrix, phase encoding direction anterior to posterior, GRAPPA acceleration factor = 2, fat-sat fat suppression). Forty slices covering the entire brain were acquired with an in-plane voxel resolution of 3.0 × 3.0 and a slice thickness of 3.4 mm with no gap. Slices were acquired in interleaved ascending order, and 210 functional volumes were acquired in each run, not including 3 volumes discarded by the scanner to account for T1 equilibrium effects. A gradient-echo field map was also acquired with the same slices and resolution as the functional images using a Siemens field map sequence (TR = 1000 ms, TE1 = 10 ms, TE2 =  12.45 ms, flip angle = 90°, 64 × 64 matrix).

A T1-weighted high-resolution (1 × 1 × 1 mm) image was acquired using a three-dimensional magnetization-prepared rapid acquisition gradient (MPRAGE) sequence (TR = 2530 msec, TE = 3.13 msec, flip angle = 10°, 256 × 256 matrix, phase encoding direction right to left, no fat suppression). Two hundred and eight coronal slices covering the entire brain were acquired in interleaved order with a voxel resolution of 1 × 1 × 1 mm. We also collected a T2-weighted anatomical scan (TR = 10,000 ms, TE = 88 ms, flip angle = 120°, 256 × 256 matrix) with 40 transverse slices with a voxel resolution of 0.82 × 0.82 × 3.5 mm that was reviewed by a radiologist to rule out incidental findings.

### fMRI Data Analysis

fMRI analysis was performed using FEAT version 6.00, FSL’s fMRI analysis tool (FMRIB’s Software Library http://fsl.fmrib.ox.ac.uk/fsl/fslwiki/) and other FSL tools from FSL version 5.0.8. Data were first corrected for magnetic field inhomogeneities using the field maps acquired for each subject and FSL’s FUGUE utility for geometrically unwarping EPIs, unwarping in the anterior-posterior (−y) direction with a 10% signal loss threshold. Data were then preprocessed using standard steps in the following order: motion correction using a rigid-body alignment to the middle volume of each run, slice-timing correction using Fourier-space time-series phase-shifting, removal of skull using FSL’s BET brain extraction tool, 5 mm FWHM spatial smoothing, and highpass temporal filtering using Gaussian-weighted least-squares straight line fitting with a sigma of 60 s (corresponding to a period of 120 s). Finally, temporal autocorrelation was removed using FSL’s prewhitening algorithm before statistical modeling^27^.

The skull was removed from the T1 images using the BET brain extraction tool with a fractional intensity thresholding setting of 0.4, and specifying the voxel that represented the approximate center of the brain. We then used FLIRT to register the functional images to the skull-stripped T1-weighted MP-RAGE using its boundary-based registration (BBR) algorithm. Next, the MP-RAGE was registered to the standard MNI atlas with a 12 degrees of freedom affine transformation, and then this transformation was refined using FNIRT nonlinear registration with a warp resolution of 10 mm^28^.

Data were then analyzed within the General Linear Model using a multi-level mixed-effects design. Each component of the task (statement, challenge, and rating) was modeled by convolving the task design with a double-gamma hemodynamic response function with a phase of 0 s. The task periods were defined from stimulus onset to stimulus offset. Political and non-political trials were modeled using separate regressors, yielding six task regressors. The temporal derivative of each task regressor and six motion correction parameters were also included in the design. At the individual subject level, statistical maps were generated for each functional scan. These were then combined into individual participant-level maps in a fixed effects analysis across each subject’s four scans. Subject-level maps were then entered into a higher-level group analysis to examine group-level effects using a “mixed effects” design with FLAME1.

To explore the relationship between the degree of belief change and brain activity in response to specific statements, we also performed a whole brain item-wise analysis. In this analysis, we first modeled each lower-level run with a design that specified a single regressor for each trial’s statements and challenges. Therefore, in this design, there were 8 task regressors (4 statements and 4 challenge periods) in addition to the six motion parameters. Task periods were modeled as in the previous analysis, using the time from stimulus onset to offset convolved with a double-gamma hemodynamic response function with phase 0 s. We then computed brain-activity maps for each specific stimulus item, combining across all subjects who read that stimulus using a second-level FLAME1 “mixed effects” design to produce item-level activity maps. These item-level activity maps were then tested for correlation with the average belief change across items in a third-level FLAME1 design that included belief change as a between-items covariate.

For all whole-brain analyses, statistical thresholding was performed using FSL’s cluster thresholding algorithm to control for multiple comparisons. This algorithm uses Gaussian Random Field Theory to estimate the probability of clusters of a given size taking into account the smoothness of the data. We used a Z threshold of 2.3, and a cluster size probability threshold of *p *< 0.05.

In addition to whole brain analysis, we performed a region of interest (ROI) analysis focusing on *a-priori* ROIs in the amygdala and insular cortex. We chose these two ROIs because of their well-known roles in emotion and feeling. For this analyis, beta values from the GLM analysis were extracted for each subject, and averaged within each ROI. The contrast used for this analysis combined activity from the period when participants were reading all political and nonpolitical challenges to their beliefs. Because there was very little belief change for political statements, we used belief change on non-political statements as our measure of individual variability. The beta values and the average belief change scores were subjected to a Shapiro-Wilk test for normality. These values were then correlated with each participant’s average belief change score in a Pearson’s correlation. The regions of interest were defined as follows: For the amygdala, we used the Harvard-Oxford Atlas amygdala mask, thresholded at 25. For the insula, we used masks of the dorsal anterior, ventral anterior, and posterior insula defined by a study that performed a cluster analysis of functional connectivity patterns^29^.

### Followup Questionnaire

Following their participation in the fMRI portion of the study, participants were sent an on-line questionnaire asking them to indicate how strongly they agreed with each statement they had seen during their fMRI scan. The average time between a participant’s scan and completing the questionnaire was 48.36 ± 5.85 days.

## Results

### Behavioral results: Belief change

**Figure 1:**
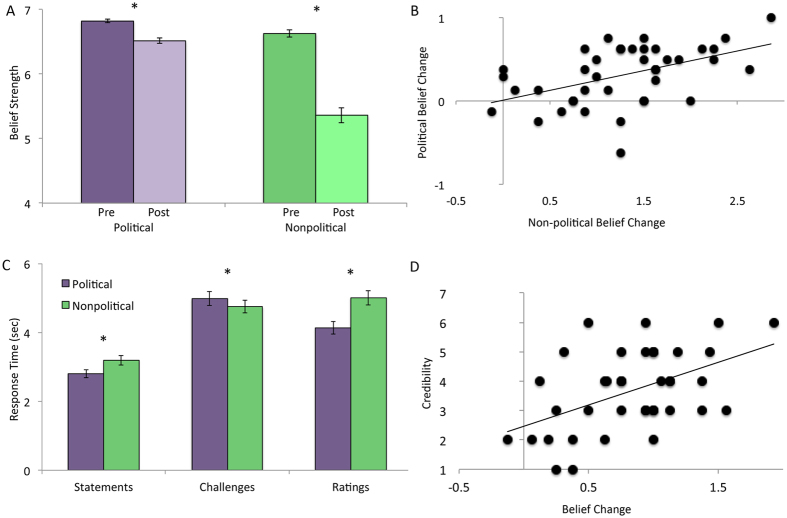
Behavioral results. (**A**) Belief strength before and after counterevidence for political and non-political trials. (**B**) Belief change on political trials correlated with belief change on non-political trials across participants. (**C**) Response times for reading statements, reading challenges, and rating the strength of belief at the end of the trial. (**D**) Increased belief change was associated with higher ratings of the credibility of the challenging arguments.

After reading the challenges to the statements, participants’ strength of belief in the statement decreased more for non-political statements than for political statements (Fig. 1A, political: 0.31 ± 0.06, non-political: 1.28 ± 0.11, t(39) = 9.76, *p *< 0.001). Additionally, the degree of belief change for political statements was correlated with the degree of belief change for non-political statements across subjects (Fig. 1B, *r* = 0.52, *p* = 0.001). In a follow-up questionnaire several weeks after the scan, participants’ strength of belief was still lower than their original strength of belief in the pre-test for both the political and non-political statements (political: 0.20 ± 0.05, t(33) = 3.62, *p* = 0.001; non-political: 0.75 ± 0.1, t(33) = 7.82, *p *< 0.001). There was no difference between belief strength in the follow-up compared to during the fMRI scan for political statements (0.12 ± 0.06, t(33) = 1.83, *p* = 0.076), however there was a difference between belief strength in the follow-up compared to during the fMRI scan for non-political statements (0.51 ± 0.13, t(33) = 4.07, *p *< 0.001). When separated by statement, average belief change across the 40 participants varied from 0.07 (abortion) to 0.32 (Thomas Edison). Generally, political statements showed the smallest degree of belief change.

### Behavioral results: Response Times

On average, participants responded that they had read and understood the statements faster for the political statements than for the non-political statements (political: 2.80 ± 0.12 seconds, non-political: 3.19 ± 0.14 seconds, t(39) = −4.21, *p *< 0.001). During the challenges, however, participants took longer to respond on the political challenges than the non-political challenges (political: 4.98 ± 0.20 seconds, non-political: 4.74 ± 0.18 seconds, t(39) = 4.02, *p *< 0.001). When participants were asked to rate their belief in the statement following the challenges, their ratings for political statements were faster than for non-political statements (political: 4.14 ± 0.18 seconds, non-political: 5.01 ± 0.22 seconds, t(39) = −5.50, *p *< 0.001). Response time data are shown in Fig. 1C.

### Behavioral Results: Credibility and Challenging Ratings

Following the fMRI scan, participants rated how credible they found the challenges in general, and how challenging they were to their beliefs. On average, participants rated credibility of the challenges at 3.63 ± 0.21 and how challenging they were to their beliefs at 3.92 ± 0.20 on a scale from 1 to 7 where 1 was not credible/challenging at all and 7 was very credible/challenging.

The more credible participants found the challenges, the higher degree of belief change they showed during the fMRI test both for the non-political statements (*r* = 0.55, *p *< 0.001) and for all statements (Fig. 1D, *r* = 0.49, *p *< 0.001). Additionally, those participants who found the challenges to be more challenging to their own beliefs showed a greater degree of change in their beliefs overall (*r* = 0.321, *p *< 0.05).

### Brain Imaging Results

Using the General Linear Model, we contrasted brain activity while participants were considering the challenges compared to resting baseline. Reading and responding to the challenges resulted in widespread activity throughout the brain, including medial and lateral occipital cortices, the inferior parietal lobule, the medial parietal cortex, large areas of the temporal lobes, the lateral frontal cortex in both hemispheres, the medial frontal cortex, the striatum, and the cerebellum (Fig. S2).

**Figure 2:**
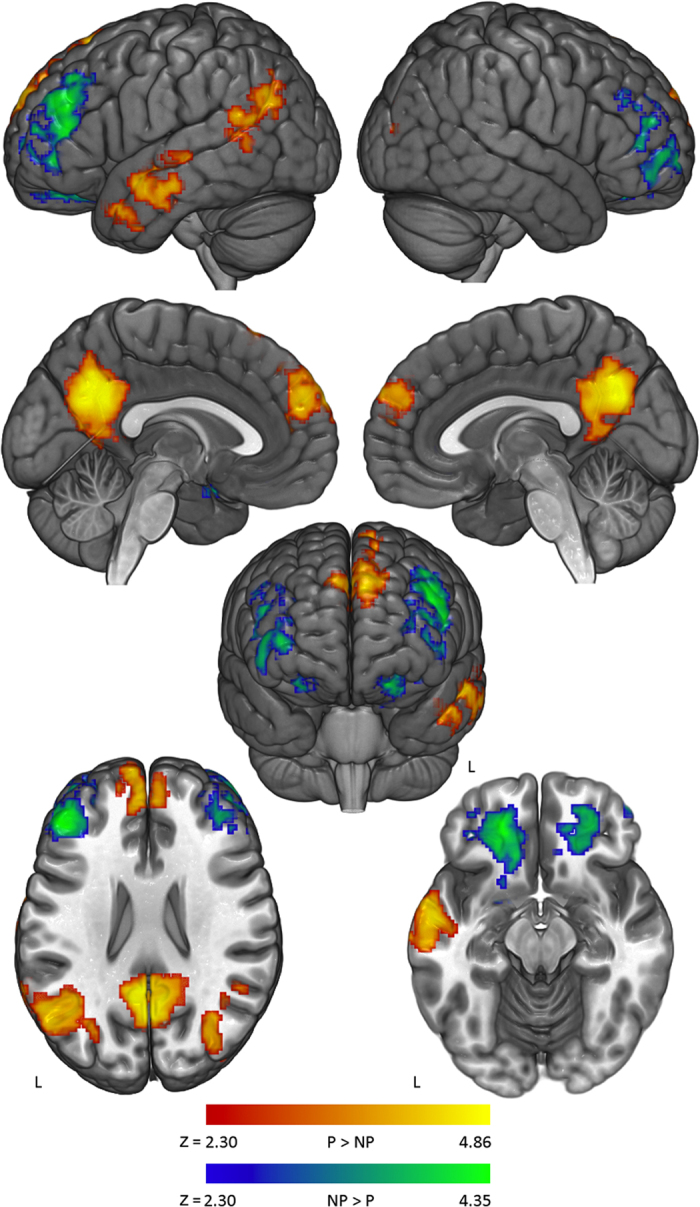
Brain activation during challenges to political vs. non-political beliefs. In red/yellow, brain regions that showed increased signal while processing challenges to political beliefs (P > NP). In blue/green, brain regions that showed increased signal during challenges to non-political beliefs (NP > P).

The main contrast of interest compared brain activity during challenges in political versus non-political beliefs (Fig. 2, Table S1). Processing challenges to political beliefs was associated with relatively increased activity in regions of the DMN, including the precuneus, the posterior cingulate cortex, the medial prefrontal cortex, the inferior parietal lobe, and the anterior temporal lobe. The opposite contrast showed increased activity in the dorsolateral prefrontal cortices and the orbitofrontal cortices bilaterally for non-political challenges compared with political challenges.

**Figure 3:**
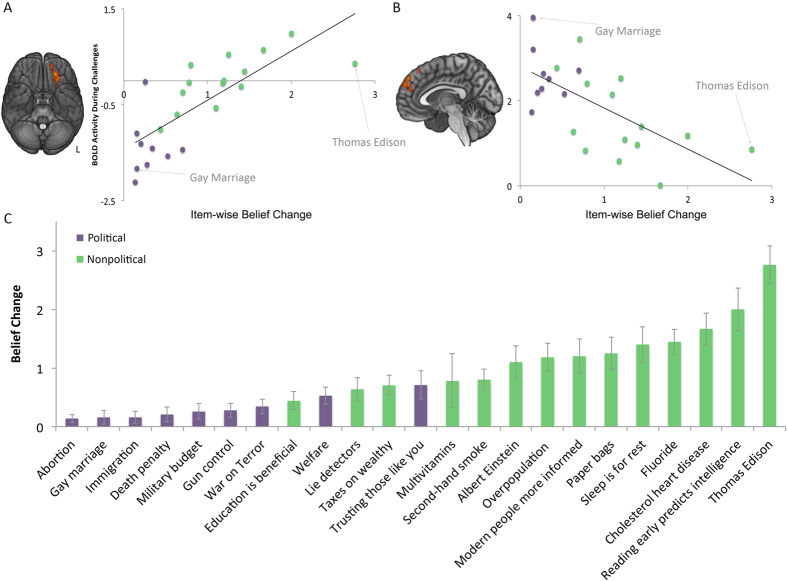
The relationship between belief change and brain activity. (**A**) Across all stimuli, BOLD signal in orbitofrontal cortex during challenges correlated positively with amount of belief change. (**B**) Across all stimuli, BOLD signal in dorsomedial prefrontal cortex correlated negatively with amount of belief change. Scatter plots visualize the relationship found in the peak voxels in each region. (**C**) Stimulus items in order of average belief change.

To explore the relationship between the degree of belief change and brain activity, we next asked whether item-level differences in belief persistence were related to brain activity while reading challenges to those items (Fig. 3C depicts the degree of belief change across the different statements). Two brain regions showed activity that significantly correlated with belief persistence across items (Fig. 3A,B, Table S2). Signal levels in the left OFC correlated negatively with resistance to belief change, whereas a region in the left dorsomedial prefrontal cortex (DMPFC) correlated positively. Figure 3 shows how individual items corresponded to signal levels. We note that the scatter plots in the figure do not represent an independent statistical analysis and are instead included as a visualization of the pattern of activity in these regions.

**Figure 4:**
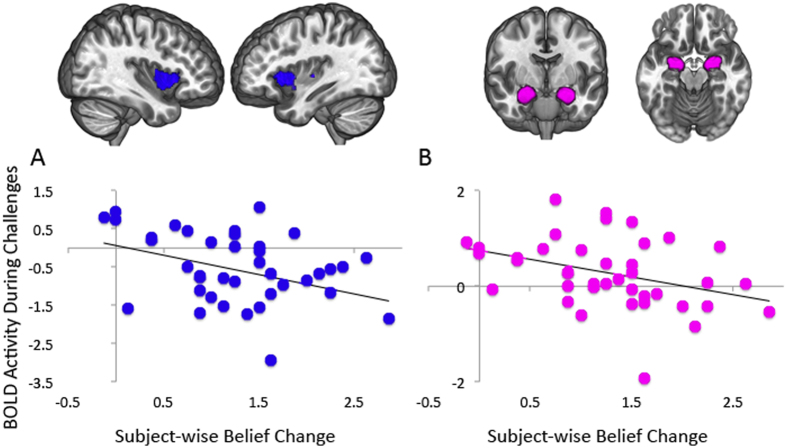
BOLD signal during challenges correlated with belief change across participants in (**A**) dorsal anterior insular cortex and (**B**) amygdala, from region of interest analysis.

In addition to item-wise correlations, we looked at differences across individuals to examine how individual variations in belief persistence related to brain activity while evaluating counterevidence using a region-of-interest approach. Given that there was very little variability in subjects’ response to political challenges, we used belief change from non-political challenges to characterize individual variability in belief-change resistance. In a region of interest analysis, we examined signal in three subregions of the insular cortex and in the amygdala complex. A Shapiro-Wilk test failed to reject the null hypothesis that the signal change data from the ROIs and the average belief change data came from normal distributions. We found that individuals who showed greater resistance to changing their beliefs showed greater activity in the dorsal anterior insular cortex (*r* = 0.35, *p *< 0.05) and in the amygdala (*r* = 0.364, *p *< 0.05) when processing challenges to their beliefs (Fig. 4). Signal in the posterior insula (*r* = −0.293, *p* = 0.066) and ventral anterior insula (*r* = −0.255, *p* = 0.112) did not significantly correlate with belief change.

## Discussion

As predicted, participants were especially resistant to arguments against their political beliefs. Post-challenge belief strength was reduced for both political and non-political statements, indicating that the counterevidence did, at least temporarily, affect reported belief strength. However, the change was significantly greater for non-political beliefs. Follow-up questionnaires completed weeks later showed that reduced belief strength persisted for the non-political beliefs.

Defending one’s beliefs against challenging evidence is a form of internally directed cognition, involving both disconnection from the externally presented evidence and a search through memory for relevant counterarguments^15^. Given the personal importance of political beliefs for the subjects enrolled in this study, we expected our stimuli to evoke cognition related to social identity. Increased activation of the DMN during challenges to political beliefs is, therefore, consistent with what is known about these structures^20^,^22^. In previous work, we have found increased signal in the precuneus, the inferior parietal cortex, and the medial prefrontal cortex when participants with strong beliefs about religion evaluated religious beliefs compared with nonreligious beliefs^30^. This is consistent with the idea that the DMN is recruited when thinking about deeply held beliefs. A related fMRI study recently found that many of the same regions correlated with certainty when people evaluated belief statements^31^. Similarly, we have found this network to be preferentially activated when people read stories that appeal to values that are perceived as strongly held and non-negotiable (i.e. “protected values”) compared to reading similar stories in which protected values are not perceived^32^. In that paper, we argue that the DMN is anatomically equipped to function as a high-level coordinator across sensory, motor, and memory domains, giving it an important role in the search and integration process that is required to create coherent meaning. Activation of the posterior medial parietal cortex is also consistent with another study of motivated reasoning in politics^10^, which found increased activity here when people processed threatening information about their candidate compared with exculpatory information.

Several alternate explanations of this result appear to be ruled out by our data. One possibility is that DMN activation here represents increased time “off-task” during the stimulation periods, since this network is known to increase in activity during mind-wandering^33^. However, our response time data argue against this interpretation, as participants took longer to respond to political challenges compared with non-political challenges. Another alternative is that stimuli in the political condition may have been more likely to describe social or mental phenomena. This explanation is not supported by our analysis of the stimuli that showed similar frequency of social and cognitive words, with slightly higher frequency in the non-political stimuli.

The opposite contrast (non-political versus political) revealed increased signal in the orbitofrontal cortex (OFC) and the dorsolateral prefrontal cortex (DLPFC). Interestingly, the OFC and the DLPFC appear critical to various forms of cognitive flexibility. Both regions, but particularly the OFC, are activated when previously learned behavioral contingencies must be overridden in favor of new ones, as in the case of reversal learning^34^,^35^,^36^. Relatedly, decreased activation in the DLPFC and the OFC is associated with cognitive inflexibility, such as that found in obsessive-compulsive disorder or addiction^37^,^38^,^39^. Our data suggest that the function of these brain regions in adjusting learned associations may be important for the process of changing one’s beliefs in response to counterevidence.

In addition to showing greater activity for non-political trials, signal in the OFC showed an item-wise correlation with belief change, again consistent with the OFC’s role in cognitive flexibility. Another brain region, the dorsomedial prefrontal cortex (DMPFC), showed the opposite relationship, correlating positively with resistance to belief change. Increased activity in the DMPFC during challenges to more firmly held beliefs may relate to the role of this region in regulating negative affect^40^. The DMPFC is one of the most frequently activated regions during cognitive reappraisal^41^, an emotion regulation strategy in which a stimulus’s meaning is deliberately reinterpreted to reduce affect.

We found that individual differences in resistance to belief change correlated with activity in the insular cortex and in the amygdala. The insular cortex, which receives projections from interoceptive neural systems that monitor the internal state of the body, is believed to be important for the generation of emotions and feelings^42^. The anterior insula, in particular, is implicated in the process of integrating affective information into decision-making^43^. In addition to reflecting the strength of subjective feeling states in general^44^, the anterior insula is activated by specific feelings that people are likely to encounter when their core beliefs are challenged, including perceptions of threat^45^, uncertainty^46^, and anxiety^47^, and has been implicated in imaging studies of politics during motivated reasoning^10^ and viewing faces of opposing political candidates^48^. The insula is also closely connected anatomically and functionally to the amygdala^49^, whose role in responding to emotionally salient stimuli is well established^50^. While recent data have shown that it signals the emotional value of a wide variety of experiences, the amygdala is especially sensitive to fearful and threatening stimuli^51^. One interpretation of these activations in the context of our study is that these structures are signaling threats to deeply held beliefs in the same way they might signal threats to physical safety.

The amygdala also plays an important role in social judgments, particularly in assessing trustworthiness. Patients with amygdala lesions show increased trust of strangers^52^, and functional imaging has revealed greater activity in response to faces that are rated as untrustworthy^53^. Other studies have found the amygdala to be directly involved in detecting deceit^54^. In the present study, the participants were presented with information and engaged in evaluating its trustworthiness. Indeed, those who rated the counterevidence as less credible were less likely to change their beliefs. Increased amygdala activity, then, may be associated with increased skepticism of the material and could be an important neural signal of the persuasive potential of information. The relationship between belief-change resistance and activity in the insular cortex and the amygdala supports the role of emotion in this process and aligns with behavioral studies that have found correlations between negative affect and resistance to changes in attitude^11^. Also, Howlett and Paulus recently found that insula activity while evaluating the truth of propositions correlated with increased certainty in the truth or falsity of those propositions^31^.

We note that while activity in the amygdala and insula was correlated across individuals with belief persistence, we did not find these structures to be activated in general within the group while considering the challenges; nor did these structures appear in our direct comparison of political and non-political challenges. There are several possible explanations for this pattern of results. First, the effect may only show in those individuals who resist belief change very strongly, and therefore does not appear in the group on average. Second, it appears that greater belief change is associated with activity levels in the insula that are suppressed below resting baseline, as opposed to activity levels that are enhanced above resting baseline in highly resistant individuals. Speculatively, this could result from a successful suppression of threat-related insula activity in belief-changers.

We now turn to a discussion of the limitations of this study. First, belief change is intentionally confounded with belief content: as predicted, participants were more likely to change their beliefs for non-political statements than for political statements. Thus, brain regions that show correlations with belief change across statements may be sensitive to qualities related to the political content of the beliefs or their challenges rather than reflecting belief change per se. Indeed, these regions appear in the direct contrast between processing political and non-political challenges. However, our earlier neuroimaging studies of belief vs. disbelief suggest a content-independent role for many of the involved structures^30^,^55^. Nevertheless, there are likely to be other notable differences between considering political and non-political beliefs. For example, given that our participants had strong political identification, they also may be more knowledgeable about the political issues referenced by our stimuli than about the non-political issues. Relatedly, our participants are likely to have experience with challenges to their political beliefs and may already have prepared counter-arguments against these challenges. Indeed, self-related activations in the DMN may be found as a result of the underlying role these structures play in memory retrieval, particularly in the posterior parietal nodes^56^,^57^.

Another potential difference between political and non-political beliefs is that political beliefs tend to be more prescriptive; that is, they imply a specific policy, while the non-political beliefs tend to be more straightforward statements of fact (although some do imply a personal policy, e.g. “Taking a multivitamin improves one’s health”). As such, the non-political beliefs might be considered to be more amenable to empirical evaluation. Howlett and Paulus recently made a related distinction between testable vs. non-testable beliefs, finding that the process of evaluating the truth of these two different kinds of propositions engaged several different brain structures (as well as some in common)^31^. However, in that study the posterior parietal cortex was more active for testable compared with non-testable beliefs, and so at least in that regard, a mapping between “political” and “non-testable” across our two studies does not seem to hold. Instead, our “political” condition and their “testable” condition may elicit greater depth of processing than their counterparts.

While we attempted to make the challenges to the political statements as strong as possible, we also cannot rule out the possibility that the non-political challenges were somehow inherently more persuasive. Both categories involved exaggerations or distortions of the truth, but our political participants may have been more likely to identify these distortions for the political issues, especially if they were more familiar with these issues. It is also possible that people are generally more distrusting of political arguments if they have come to expect motivated reasoning on the part of others in this domain. We did find that participants who rated the challenges as more credible were more likely to change their minds, and it is well known that source credibility influences persuasion^58^. However, very few of our stimuli were attributed to specific sources. We view these subjective credibility ratings as inseparable from how persuaded participants were by the arguments, and therefore did not intend to equate this rating across the political and non-political categories.

Given that all of our participants were strong liberals, it is not clear how well these results would generalize to conservatives, or to people with less polarized beliefs. Several studies have found structural or functional differences between the brains of conservatives and liberals^59^,^60^. One specifically relevant difference is the finding of larger right amygdala volume in conservatives^61^. Relatedly, conservatism tends to be associated with increased threat avoidance^62^. In our data, activity in the amygdala when beliefs were challenged was associated with increased resistance to belief change. We note that while our participants expressed trait liberalism, in the context of this experiment they were motivated to conserve their specific beliefs against a direct threat.

While extreme cognitive inflexibility in the face of new information has the potential to be problematic, we do not intend to suggest that the motivation to protect one’s beliefs is necessarily maladaptive. Indeed, there is likely some benefit to be gained from providing a degree of protection to useful beliefs, and changing one’s mental models without sufficient reason would cause problems of its own.

Our results show that when people are confronted with challenges to their deeply held beliefs, they preferentially engage brain structures known to support stimulus-independent, internally directed cognition. Our data also support the role of emotion in belief persistence. Individual differences in persuasion were related to differences in activity within the insular cortex and the amygdala—structures crucial to emotion and feeling. The brain’s systems for emotion, which are purposed toward maintaining homeostatic integrity of the organism^63^, appear also to be engaged when protecting the aspects of our mental lives with which we strongly identify, including our closely held beliefs.

## Additional Information

**How to cite this article**: Kaplan, J. T. *et al*. Neural correlates of maintaining one’s political beliefs in the face of counterevidence. *Sci. Rep.* **6**, 39589; doi: 10.1038/srep39589 (2016).

**Publisher's note:** Springer Nature remains neutral with regard to jurisdictional claims in published maps and institutional affiliations.

## Supplementary Material

Supplementary Materials

## References

[b1] Ahluwalia R. Examination of psychological processes underlying resistance to persuasion. J Consum Res 27, 217–232, doi: 10.1086/314321 (2000).

[b2] Jacks J. Z. & Devine P. G. Attitude importance, forewarning of message content, and resistance to persuasion. Basic Appl Soc Psych 22, 19–29, doi: 10.1207/S15324834basp2201_3 (2000).

[b3] Pomerantz E. M., Chaiken S. & Tordesillas R. S. Attitude strength and resistance processes. J Pers Soc Psychol 69, 408–419 (1995). 7562388 10.1037//0022-3514.69.3.408

[b4] Kunda Z. The case for motivated reasoning. Psychological bulletin 108, 480–498 (1990). 2270237 10.1037/0033-2909.108.3.480

[b5] Lord C. G., Ross L. & Lepper M. R. Biased Assimilation and Attitude Polarization - Effects of Prior Theories on Subsequently Considered Evidence. J Pers Soc Psychol 37, 2098–2109, doi: 10.1037/0022-3514.37.11.2098 (1979).

[b6] Munro G. D. . Biased assimilation of sociopolitical arguments: Evaluating the 1996 US presidential debate. Basic Appl Soc Psych 24, 15–26, doi: 10.1207/153248302753439038 (2002).

[b7] Nyhan B., Reifler J., Richey S. & Freed G. L. Effective messages in vaccine promotion: a randomized trial. Pediatrics 133, e835–842, doi: 10.1542/peds.2013-2365 (2014). 24590751

[b8] Nyhan B. & Reifler J. When Corrections Fail: The Persistence of Political Misperceptions. Polit Behav 32, 303–330, doi: 10.1007/s11109-010-9112-2 (2010).

[b9] Falk E. B. . The neural correlates of persuasion: a common network across cultures and media. J Cogn Neurosci 22, 2447–2459, doi: 10.1162/jocn.2009.21363 (2010). 19925175 PMC3025286

[b10] Westen D., Blagov P. S., Harenski K., Kilts C. & Hamann S. Neural bases of motivated reasoning: an FMRI study of emotional constraints on partisan political judgment in the 2004 U.S. Presidential election. J Cogn Neurosci 18, 1947–1958, doi: 10.1162/jocn.2006.18.11.1947 (2006). 17069484

[b11] Zuwerink J. R. & Devine P. G. Attitude importance and resistance to persuasion: It’s not just the thought that counts. J Pers Soc Psychol 70, 931–944, doi: 10.1037/0022-3514.70.5.931 (1996).

[b12] Harmon-Jones E. In Emotions and beliefs (eds N. H. Frijda, Manstead A. R. S. & Bem S.) 185–211 (Cambridge University Press, 2000).

[b13] Munro G. D. The Scientific Impotence Excuse:Discounting Belief-Threatening Scientific Abstracts. J Appl Soc Psychol 40, 579–600 (2010).

[b14] Burris C. T., Harmon-Jones E. & Tarpley W. R. “By faith alone”: Religious agitation and cognitive dissonance. Basic Appl Soc Psych 19, 17–31 (1997).

[b15] Jacks J. Z. & Cameron K. A. Strategies for resisting persuasion. Basic Appl Soc Psych 25, 145–161, doi: 10.1207/S15324834basp2502_5 (2003).

[b16] Fleming M. & Petty R. In Attitudes, behavior, and social context: The role of norms and group membership (eds Deborah Terry & Michael Hogg) 171–199 (Lawrence Erlbaum Associates, 2000).

[b17] Cohen G. L. Party over policy: The dominating impact of group influence on political beliefs. J Pers Soc Psychol 85, 808–822, doi: 10.1037/0022-3514.85.5.808 (2003). 14599246

[b18] Conover P. J. The Role of Social-Groups in Political Thinking. Brit J Polit Sci 18, 51–76 (1988).

[b19] Unsworth K. L. & Fielding K. S. It’s political: How the salience of one’s political identity changes climate change beliefs and policy support. Global Environ Chang 27, 131–137, doi: 10.1016/j.gloenvcha.2014.05.002 (2014).

[b20] Buckner R. L., Andrews-Hanna J. R. & Schacter D. L. The brain’s default network: anatomy, function, and relevance to disease. Annals of the New York Academy of Sciences 1124, 1–38, doi: 10.1196/annals.1440.011 (2008). 18400922

[b21] Raichle M. E. & Snyder A. Z. A default mode of brain function: a brief history of an evolving idea. Neuroimage 37, 1083–1090, discussion 1097–1089, doi: 10.1016/j.neuroimage.2007.02.041 (2007). 17719799

[b22] Whitfield-Gabrieli S. . Associations and dissociations between default and self-reference networks in the human brain. Neuroimage 55, 225–232, doi: 10.1016/j.neuroimage.2010.11.048 (2011). 21111832

[b23] Northoff G. . Self-referential processing in our brain–a meta-analysis of imaging studies on the self. Neuroimage 31, 440–457, doi: 10.1016/j.neuroimage.2005.12.002 (2006). 16466680

[b24] Flesch R. A New Readability Yardstick. J Appl Psychol 32, 221–233, doi: 10.1037/h0057532 (1948). 18867058

[b25] Linguistic inquiry and word count: LIWC (Liwc.Net, Austin, TX, 2007).

[b26] Dehghani M. . TACIT: An open-source text analysis, crawling, and interpretation tool. *Social Science Research Network* (2015). 10.3758/s13428-016-0722-4 26944580

[b27] Woolrich M. W., Ripley B. D., Brady M. & Smith S. M. Temporal autocorrelation in univariate linear modeling of FMRI data. Neuroimage 14, 1370–1386, doi: 10.1006/nimg.2001.0931 (2001). 11707093

[b28] Andersson J., Jenkinson M. & Smith S. Non-linear registration, aka Spatial normalisation. *FMRIB technical report TR07JA2* (2007).

[b29] Deen B., Pitskel N. B. & Pelphrey K. A. Three systems of insular functional connectivity identified with cluster analysis. Cereb Cortex 21, 1498–1506, doi: 10.1093/cercor/bhq186 (2011). 21097516 PMC3116731

[b30] Harris S. . The Neural Correlates of Religious and Nonreligious Belief. Plos One 4, doi: 10.1371/Journal.Pone.0007272 (2009). PMC2748718 19794914

[b31] Howlett J. R. & Paulus M. P. The neural basis of testable and non-testable beliefs. Plos One 10, e0124596, doi: 10.1371/journal.pone.0124596 (2015). 25942019 PMC4420500

[b32] Kaplan J. T. . Processing Narratives Concerning Protected Values: A Cross-Cultural Investigation of Neural Correlates. Cereb Cortex, doi: 10.1093/cercor/bhv325 (2016). 26744541

[b33] Smallwood J. & Schooler J. W. The science of mind wandering: empirically navigating the stream of consciousness. Annu Rev Psychol 66, 487–518, doi: 10.1146/annurev-psych-010814-015331 (2015). 25293689

[b34] Ghahremani D. G., Monterosso J., Jentsch J. D., Bilder R. M. & Poldrack R. A. Neural components underlying behavioral flexibility in human reversal learning. Cereb Cortex 20, 1843–1852, doi: 10.1093/cercor/bhp247 (2010). 19915091 PMC2901019

[b35] Schoenbaum G., Roesch M. R., Stalnaker T. A. & Takahashi Y. K. A new perspective on the role of the orbitofrontal cortex in adaptive behaviour. Nat Rev Neurosci 10, 885–892, doi: 10.1038/nrn2753 (2009). 19904278 PMC2835299

[b36] Xue G. . Common neural mechanisms underlying reversal learning by reward and punishment. Plos One 8, e82169, doi: 10.1371/journal.pone.0082169 (2013). 24349211 PMC3859585

[b37] Gu B. M. . Neural correlates of cognitive inflexibility during task-switching in obsessive-compulsive disorder. Brain 131, 155–164, doi: 10.1093/brain/awm277 (2008). 18065438

[b38] Verdejo-Garcia A. . Neural substrates of cognitive flexibility in cocaine and gambling addictions. Br J Psychiatry, doi: 10.1192/bjp.bp.114.152223 (2015). 26045346

[b39] Chamberlain S. R. . Orbitofrontal dysfunction in patients with obsessive-compulsive disorder and their unaffected relatives. Science 321, 421–422, doi: 10.1126/science.1154433 (2008). 18635808

[b40] Silvers J. A., Wager T. D., Weber J. & Ochsner K. N. The neural bases of uninstructed negative emotion modulation. Soc Cogn Affect Neurosci 10, 10–18, doi: 10.1093/scan/nsu016 (2015). 24493847 PMC4994839

[b41] Buhle J. T. . Cognitive reappraisal of emotion: a meta-analysis of human neuroimaging studies. Cereb Cortex 24, 2981–2990, doi: 10.1093/cercor/bht154 (2014). 23765157 PMC4193464

[b42] Damasio A. & Carvalho G. B. The nature of feelings: evolutionary and neurobiological origins. Nat Rev Neurosci 14, 143–152, doi: 10.1038/nrn3403 (2013). 23329161

[b43] Critchley H. D. Neural mechanisms of autonomic, affective, and cognitive integration. J Comp Neurol 493, 154–166, doi: 10.1002/cne.20749 (2005). 16254997

[b44] Craig A. D. How do you feel–now? The anterior insula and human awareness. Nat Rev Neurosci 10, 59–70, doi: 10.1038/nrn2555 (2009). 19096369

[b45] Wiech K. . Anterior insula integrates information about salience into perceptual decisions about pain. J Neurosci 30, 16324–16331, doi: 10.1523/JNEUROSCI.2087-10.2010 (2010). 21123578 PMC6634837

[b46] Lamm C. & Singer T. The role of anterior insular cortex in social emotions. Brain Struct Funct 214, 579–591, doi: 10.1007/s00429-010-0251-3 (2010). 20428887

[b47] Critchley H. D., Wiens S., Rotshtein P., Ohman A. & Dolan R. J. Neural systems supporting interoceptive awareness. Nat Neurosci 7, 189–195, doi: 10.1038/nn1176 (2004). 14730305

[b48] Kaplan J. T., Freedman J. & Iacoboni M. Us versus them: Political attitudes and party affiliation influence neural response to faces of presidential candidates. Neuropsychologia 45, 55–64, doi: 10.1016/j.neuropsychologia.2006.04.024 (2007). 16764897

[b49] Baur V., Hanggi J., Langer N. & Jancke L. Resting-state functional and structural connectivity within an insula-amygdala route specifically index state and trait anxiety. Biol Psychiatry 73, 85–92, doi: 10.1016/j.biopsych.2012.06.003 (2013). 22770651

[b50] Adolphs R., Tranel D., Damasio H. & Damasio A. Impaired recognition of emotion in facial expressions following bilateral damage to the human amygdala. Nature 372, 669–672, doi: 10.1038/372669a0 (1994). 7990957

[b51] Adolphs R. Fear, faces, and the human amygdala. Curr Opin Neurobiol 18, 166–172, doi: 10.1016/j.conb.2008.06.006 (2008). 18655833 PMC2580742

[b52] Adolphs R., Tranel D. & Damasio A. R. The human amygdala in social judgment. Nature 393, 470–474, doi: 10.1038/30982 (1998). 9624002

[b53] Engell A. D., Haxby J. V. & Todorov A. Implicit trustworthiness decisions: automatic coding of face properties in the human amygdala. J Cogn Neurosci 19, 1508–1519, doi: 10.1162/jocn.2007.19.9.1508 (2007). 17714012

[b54] Grezes J., Berthoz S. & Passingham R. E. Amygdala activation when one is the target of deceit: did he lie to you or to someone else? Neuroimage 30, 601–608, doi: 10.1016/j.neuroimage.2005.09.038 (2006). 16257239

[b55] Harris S., Sheth S. A. & Cohen M. S. Functional neuroimaging of belief, disbelief, and uncertainty. Ann Neurol 63, 141–147, doi: 10.1002/Ana.21301 (2008). 18072236

[b56] Araujo H. F., Kaplan J., Damasio H. & Damasio A. Involvement of cortical midline structures in the processing of autobiographical information. PeerJ 2, e481, doi: 10.7717/peerj.481 (2014). 25097820 PMC4121543

[b57] Sestieri C., Corbetta M., Romani G. L. & Shulman G. L. Episodic memory retrieval, parietal cortex, and the default mode network: functional and topographic analyses. J Neurosci 31, 4407–4420, doi: 10.1523/JNEUROSCI.3335-10.2011 (2011). 21430142 PMC3098040

[b58] Pornpitakpan C. The persuasiveness of source credibility: A critical review of five decades’ evidence. J Appl Soc Psychol 34, 243–281, doi: 10.1111/j.1559-1816.2004.tb02547.x (2004).

[b59] Amodio D. M., Jost J. T., Master S. L. & Yee C. M. Neurocognitive correlates of liberalism and conservatism. Nat Neurosci 10, 1246–1247, doi: 10.1038/nn1979 (2007). 17828253

[b60] Zamboni G. . Individualism, conservatism, and radicalism as criteria for processing political beliefs: a parametric fMRI study. Soc Neurosci 4, 367–383, doi: 10.1080/17470910902860308 (2009). 19562629

[b61] Kanai R., Feilden T., Firth C. & Rees G. Political orientations are correlated with brain structure in young adults. Curr Biol 21, 677–680, doi: 10.1016/j.cub.2011.03.017 (2011). 21474316 PMC3092984

[b62] Jost J. T. & Amodio D. M. Political ideology as motivated social cognition: Behavioral and neuroscientific evidence. Motiv Emotion 36, 55–64, doi: 10.1007/s11031-011-9260-7 (2012).

[b63] Damasio A. R. Self comes to mind: constructing the conscious brain 1st edn (Pantheon Books, 2010).

