# Supplementary material for: Neural correlates of maintaining one’s political beliefs in the face of counterevidence
Source: Sci Rep. 2016 Dec 23;6:39589. doi: 10.1038/srep39589 (PMC5180221; doi:10.1038/srep39589)
Supplement: Supplementary Materials [file srep39589-s1.pdf]

Neural correlates of maintaining one's political beliefs in the face of counterevidence

Authors: Jonas T. Kaplan, Sarah I. Gimbel, and Sam Harris

Supplementary Materials

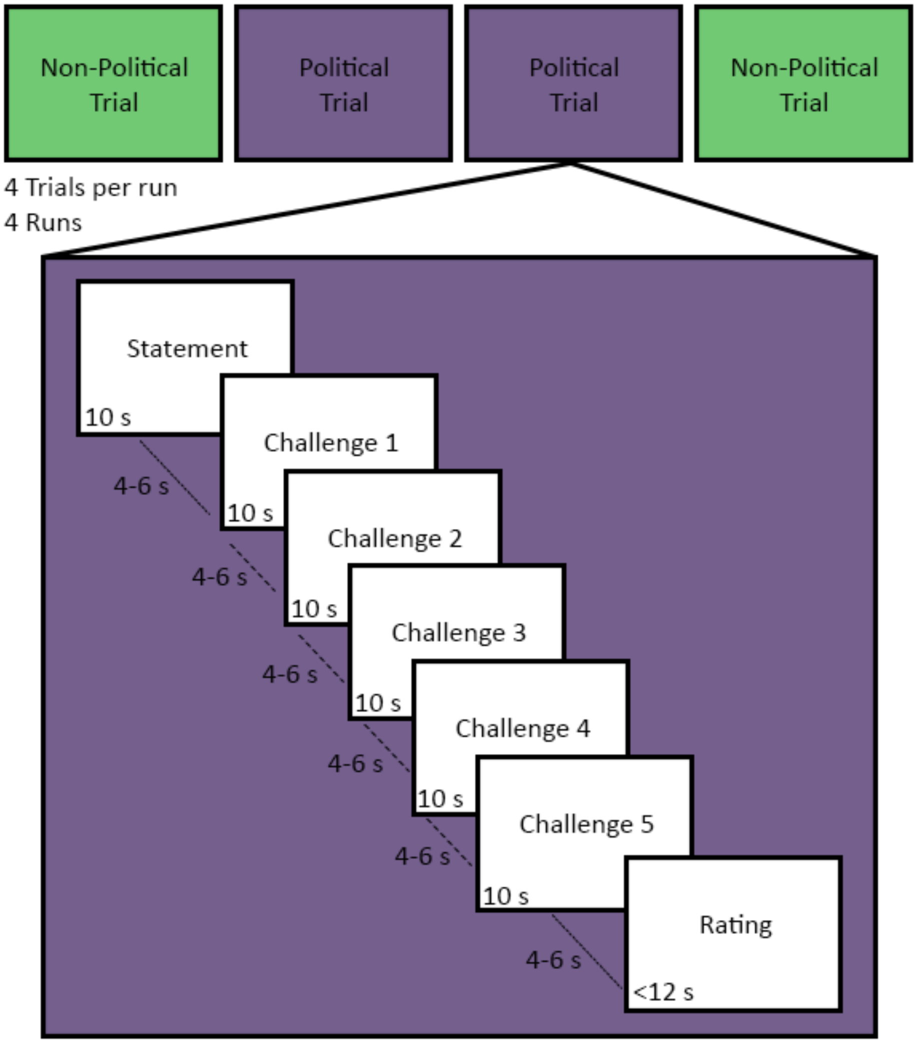

Fig. S1: Experimental design. In each of 4 fMRI scans participants were presented with 4 trials, consisting of 2 political and 2 nonpolitical stimulus sets. In each trial, participants first saw a statement that described a belief they had previously endorsed. Next, they were presented with five challenging statements that argued against those beliefs. Finally, they were presented with the original statement again and asked to rate the strength of their belief on a scale from 1 to 7.

Fig S2: Activation during challenge periods compared with resting baseline. Reading and contemplating the challenges led to greater activation throughout the brain when compared with resting baseline, including large portions of the frontal, temporal, and occipital lobes.

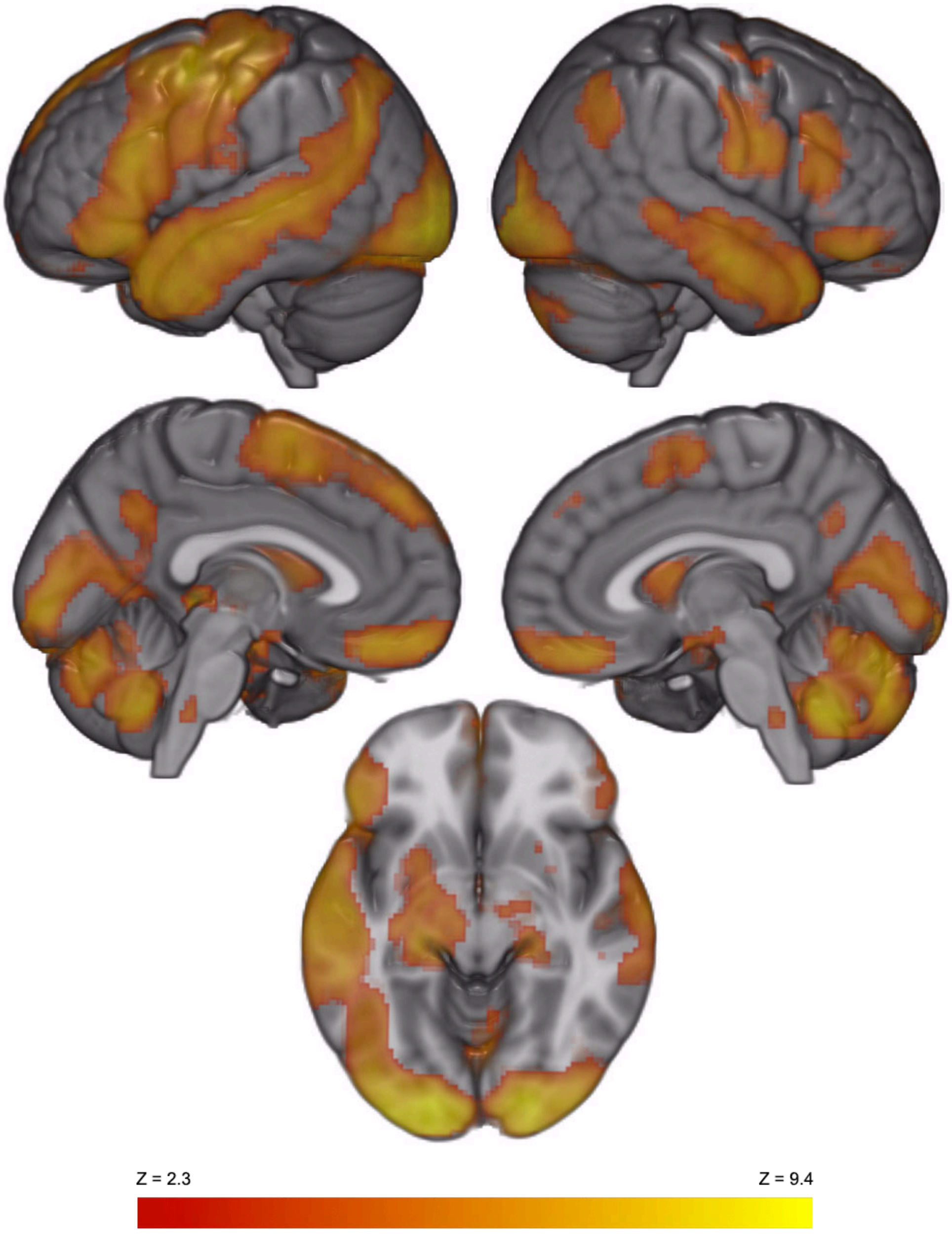

Table S1: Peak activations for Political minus Nonpolitical (P-NP) and Nonpolitical minus Political (NP-P) brain activations during challenges.

Region		Z-value	x	y	z
**P-NP**					
Precuneus	L/R	5.47	-6	-52	26
Medial Prefrontal Cortex	L/R	4.42	-8	44	44
Anterior Temporal Lobe	L	4.57	-60	-4	-16
Inferior Parietal Lobe	L	3.99	-54	-60	26
Inferior Parietal Lobe	R	3.79	30	-64	32
**NP-P**					
Dorsolateral Prefrontal Cortex	L	5.18	-44	38	22
Dorsolateral Prefrontal Cortex	R	3.66	40	48	18
Orbitofrontal Cortex	L	4.97	-22	42	-18
Orbitofrontal Cortex	R	3.88	28	40	-16

Table S2: Peak activations for correlations between belief change and signal across stimulus items

Region		Z-value	x	y	z
Orbitofrontal Cortex	L	3.95	-22	36	-18
Dorsomedial Prefrontal Cortex	R	-3.56	-8	56	40

Table S3: Political and nonpolitical statements.

Political statements	Nonpolitical statements
The U.S. should reduce its military budget.	Long term exposure to second-hand smoke is a significant health concern.
The laws regulating gun ownership in the United States should be made more restrictive.	Lowering one's consumption of foods that are high in cholesterol is a good way to prevent heart disease.
Welfare and food stamp programs offer necessary help to the poor.	People tend to feel the most trust for those who are most like them racially, culturally, economically, etc.
The U.S. "War on Terror" has been an unnecessary and ineffective response to the attacks of September 11th.	A college education usually improves a person's economic prospects.
Generally speaking, taxes on the wealthiest Americans should be increased.	Taking a daily multivitamin improves one's health.
Gay marriage should be legal in the United States.	An ability to read very early in childhood is usually a sign of intelligence.
Abortion should be legal.	Albert Einstein is generally acknowledged to be one of the greatest physicists of all time.
The U.S. should reform its immigration laws and provide a path to legal residency for illegal immigrants.	Due to increased access to information, people are generally better informed today than at any other point in history.
The death penalty should be abolished in the American judicial system.	The primary purpose of sleep is to rest the body and mind.
	Thomas Edison invented the light bulb.
	Overpopulation is a serious global concern.
	Lie detectors are generally unreliable.
	Fluoride helps to prevent tooth decay.
	Paper bags are better for the environment than plastic bags.

Table S4: Example challenge stimuli

**Political example:** The laws regulating gun ownership in the United States should be made more restrictive.	**Nonpolitical example:** Thomas Edison invented the light bulb
**Challenges**	**Challenges**
98% of gun crimes are committed with stolen guns. People who possess guns illegally are unlikely to obey new laws regulating gun ownership.	Nearly 70 years before Edison, Humphrey Davy demonstrated an electric lamp to the Royal Society.
Every year, around 100,000 law-abiding gun owners defend themselves against violent crime (more than 9 times out of 10 without firing a shot).	Edison hired a young physicist, Francis Upton, to help work on the light bulb. He had the breakthrough discovery that led to the light bulb development.
More people accidentally drown each year than have been killed in gun-related accidents since 1980.	Edison's patent for the electric light bulb was invalidated by the US Patent Office, who found that it was based on the work of another inventor.
Only half of all homicides involve guns. Ten times more people are murdered with kitchen knives each year than are killed by "assault weapons."	While Edison played an important role in popularizing and marketing the electric light bulb, most historians agree that he played a minimal role in its development.
In the last 20 years, 10 million Americans received permits to carry concealed firearms in public. The rate of all violent crime (murder, rape, assault, and robbery) dropped by 50 percent during this same period.	In 1841, decades before Edison's work, a British inventor patented the enclosed bulb design to limit oxygen and keep the burners from catching fire.
